# Atypical Periocular Dermatophytosis Caused by *Trichophyton mentagrophytes* Species Complex Misdiagnosed as Refractory Bacterial and Viral Infection

**DOI:** 10.3390/diagnostics16203307

**Published:** 2026-10-12

**Authors:** Veronica Battistella, Davide Meneghesso, Maira Zoppelletto, Alberto Meneguzzo, Silvia Visentin, Michele Borghi, Mauro Guariento, Sofia Mezzalira, Alessandra Pasinato, Luisa Bonsembiante, Mattia Pasquinucci

**Affiliations:** 1 Department of Woman’s and Child’s Health, University Hospital of Padua, 35128 Padua, Italy; 2 Department of Paediatrics, AULSS 7 Pedemontana–San Bassiano Hospital, 36061 Bassano del Grappa, Italy; mauro.guariento@aulss8.veneto.it (M.G.);; 3 Department of Microbiology, AULSS 7 Pedemontana–San Bassiano Hospital, 36061 Bassano del Grappa, Italy; 4 Department of Dermatology, AULSS 7 Pedemontana–San Bassiano Hospital, 36061 Bassano del Grappa, Italy; 5 Department of Ophthalmology, AULSS 7 Pedemontana–San Bassiano Hospital, 36061 Bassano del Grappa, Italy

**Keywords:** *Trichophyton mentagrophytes* species complex, periocular tinea, tinea incognito, preseptal cellulitis, children

## Abstract

A previously healthy 4-year-old boy presented with progressive left eyelid swelling and erythema, without reported animal exposure. Initially diagnosed as impetiginized eczema, the lesion was treated with topical antibiotic and corticosteroid therapy. Due to early clinical worsening, this was subsequently escalated with oral amoxicillin–clavulanate (50 mg/kg/day) and azithromycin (10 mg/kg/day). However, this local and systemic regimen failed and worsened the clinical presentation over a 9-day period. Inflammatory markers and blood cultures remained normal. Due to outpatient treatment failure, an initial periorbital skin and ciliary margin swab was collected on day 9 of oral antibiotics. Two days later, worsening symptoms prompted hospital admission for intravenous broad-spectrum antibiotics (ceftriaxone and clindamycin) and fluconazole. Pending culture results, suspected vesicular lesions led to empirical oral acyclovir (20 mg/kg/dose, 4 times daily); this was discontinued after 3 days when the initial swab returned negative for HSV. Ultimately, after an 8-day incubation, the outpatient swab culture yielded *Trichophyton mentagrophytes* species complex. Following microbiological identification of the dermatophyte, antifungal treatment was optimized with systemic griseofulvin and topical terbinafine, leading to complete clinical resolution. This case highlights atypical periocular dermatophytosis masquerading as a refractory bacterial and viral infection. Since direct animal exposure was not reported and phenotypic identification alone cannot distinguish between zoophilic (*T. mentagrophytes*) and anthropophilic (*T. interdigitale*) subtypes, the exact source of infection remains undetermined. In conclusion, while ‘tinea incognito’ is well documented, this report illustrates an exceptionally atypical periocular manifestation that mimicked a refractory bacterial cellulitis and simulated a herpetic infection. The novelty of this case lies in its severe clinical mimicry—including the development of chalazion-like lesions—which confounded a multidisciplinary team and led to unnecessary systemic antiviral and broad-spectrum antibiotic therapies, strongly highlighting the clinical importance of considering dermatophytosis in atypical orbital presentations.

**Figure 1:**
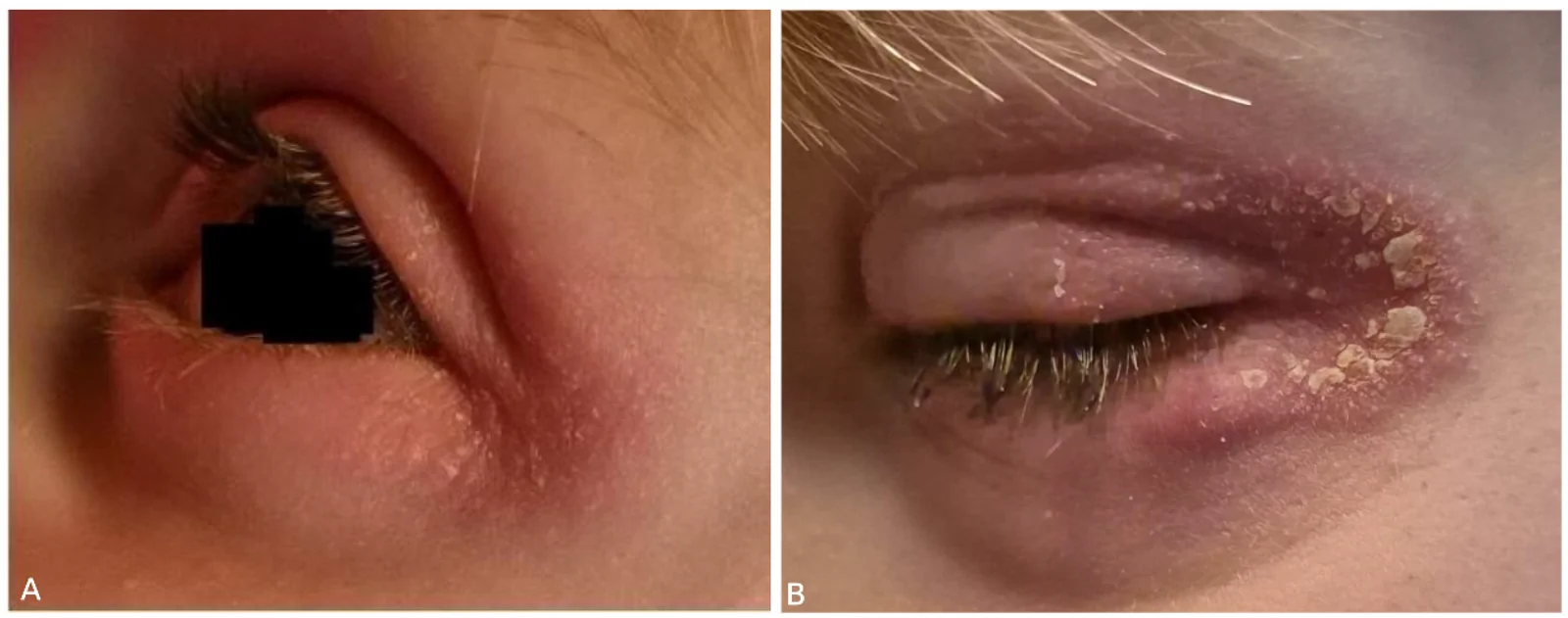
Initial clinical presentation and early progression of periocular dermatophytosis. (**A**) Initial presentation showing prominent left periocular erythema and edema involving both the upper and lower eyelids, accompanied by mild discharge. The child was afebrile and reported pruritus but no pain; ophthalmic examination revealed a spared conjunctiva, intact extraocular movements, and unimpaired visual acuity. Based on an initial misdiagnosis of impetiginized eczema—a frequent clinical pitfall, as tinea faciei commonly mimics other inflammatory dermatoses such as eczema, discoid lupus erythematosus, or rosacea, leading to delayed diagnoses—topical therapy with tobramycin/dexamethasone was initiated, alongside oral cetirizine for symptomatic relief of pruritus. (**B**) Clinical progression after three days of topical therapy (Figure S1), demonstrating worsening edema, the emergence of a mechanical ptosis and the development of prominent overlying crusts, which prompted the introduction of oral amoxicillin–clavulanate (50 mg/kg/day) and azithromycin (10 mg/kg/day) [1,2].

**Figure 2:**
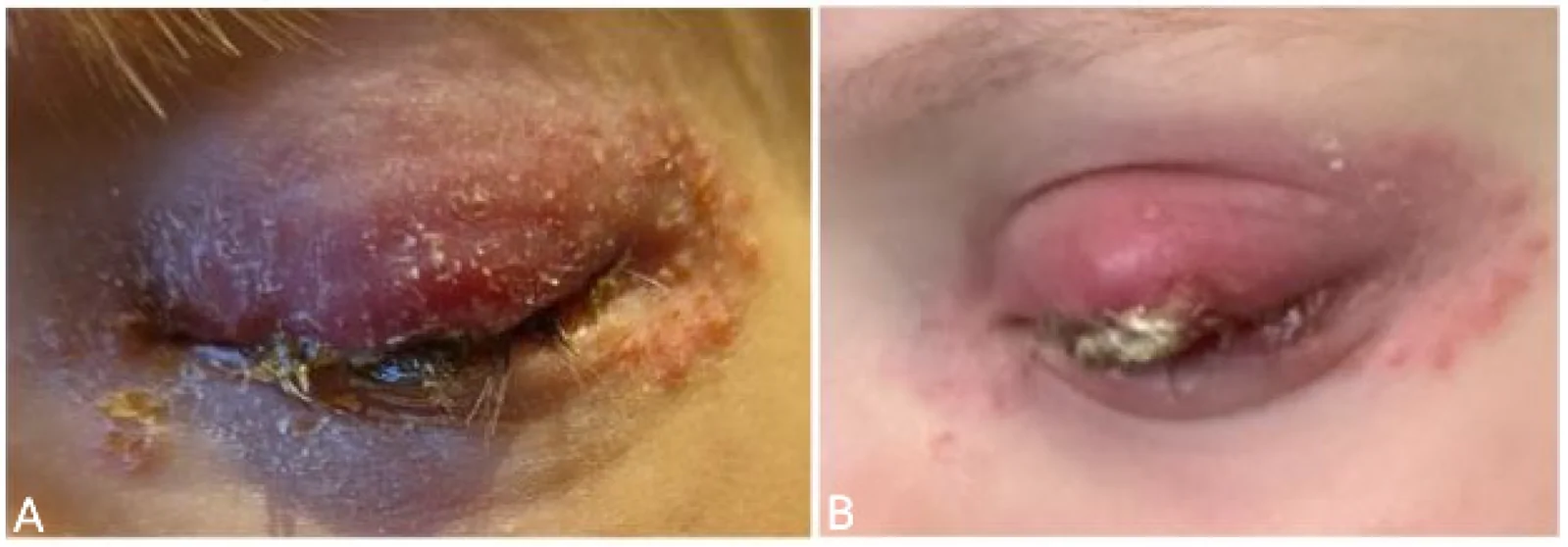
Further clinical exacerbation and manifestation of ‘tinea incognito’. (**A**) A marked worsening of the periocular lesion is observed 9 days after the initiation of systemic antibiotic therapy. The image demonstrates a prominent increase in tissue induration, severe hyperemia, and exacerbated purulent discharge, alongside clustered herpetiform pustules and vesicular lesions with mild desquamation (**B**). This atypical presentation was later interpreted as consistent with ‘tinea incognito’, potentially induced by previous topical corticosteroid use. The inappropriate use of topical corticosteroids likely suppressed local cellular immunity and altered the cutaneous microbiome, facilitating fungal proliferation while completely masking the classic annular margins of dermatophytosis [3,4,5]. The resulting prominent crusting, purulent discharge, and severe induration closely resembled the standard clinical criteria for preseptal bacterial cellulitis, making the initial diagnostic pitfall highly deceptive. Although this presentation initially prompted the empirical addition of topical mupirocin, subsequent microbiological swabs confirmed only the presence of *T. mentagrophytes* species complex. While no bacterial pathogens were isolated, these swabs were collected after 9 days of systemic and topical antibiotic therapy; therefore, a concomitant or preceding bacterial superinfection cannot be definitively excluded. Nevertheless, the clinical course underscores how steroid-modified tinea can clinically simulate a purulent bacterial infection.

**Figure 3:**
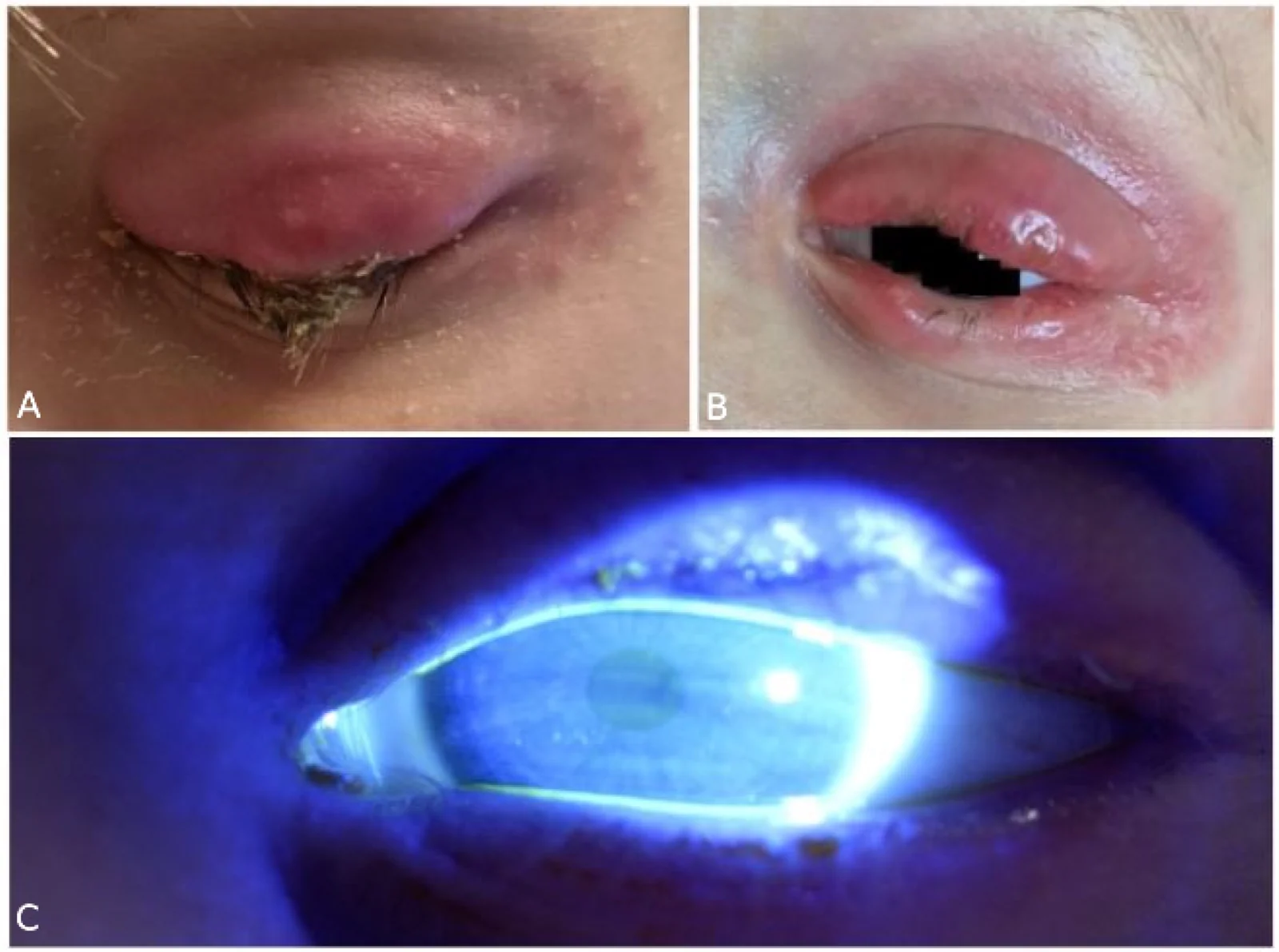
Clinical presentation at the Pediatric Emergency Department and multidisciplinary management. (**A**) The image displays the clinical status of the patient’s left eye upon presentation to our department. While a reduction in purulent discharge and crusting was noted, a concomitant worsening of erythema and upper eyelid swelling was observed, along with the appearance of multiple chalazion-like lesions along the upper ciliary margin. (**B**) Concurrently, a progressive loss of eyelashes (madarosis) became evident, signaling dermatophytic hair follicle involvement. Severe periocular presentations are unusual and can closely mimic serious bacterial or viral infections, further complicating the diagnostic workup. Due to this complex presentation, the patient was evaluated by a multidisciplinary team comprising a dermatologist, an ophthalmologist, and pediatricians. (**C**) Ophthalmological slit-lamp examination under cobalt blue illumination following fluorescein instillation demonstrated diffuse punctate epithelial keratopathy with mild fluorescein uptake. This likely represented a secondary, reactive epithelial distress due to inflammatory exposure and mechanical eyelid malposition (mechanical ptosis and lagophthalmos). Although a direct corneal scraping was not performed to definitively rule out microscopic involvement, slit-lamp examination revealed no clinical evidence of deep infectious keratitis; no dendritic patterns, stromal infiltrates, or deep ulcerations were detected. Laboratory investigations revealed a normal complete blood count and negative inflammatory markers; blood cultures were performed (subsequently yielding negative results) and a second (in-hospital) periorbital skin and ciliary margin swab was concurrently collected to rule out viral etiologies and verify the initial outpatient swab. Intravenous broad-spectrum antimicrobial therapy was initiated with ceftriaxone, clindamycin, and fluconazole. Due to the clinical suspicion of herpetic vesicles, oral acyclovir (20 mg/kg/dose, 4 times daily) was empirically added on hospital day 2. This antiviral was safely discontinued three days later, upon confirmation that both the initial outpatient swab and the repeat in-hospital swab were negative for HSV. A high index of clinical suspicion is paramount in such atypical presentations. This case highlights the complementary value of traditional fungal cultures, particularly when initial presentations are heavily modified by empirical treatments and when molecular rapid tests are not immediately available or performed.

**Figure 4:**
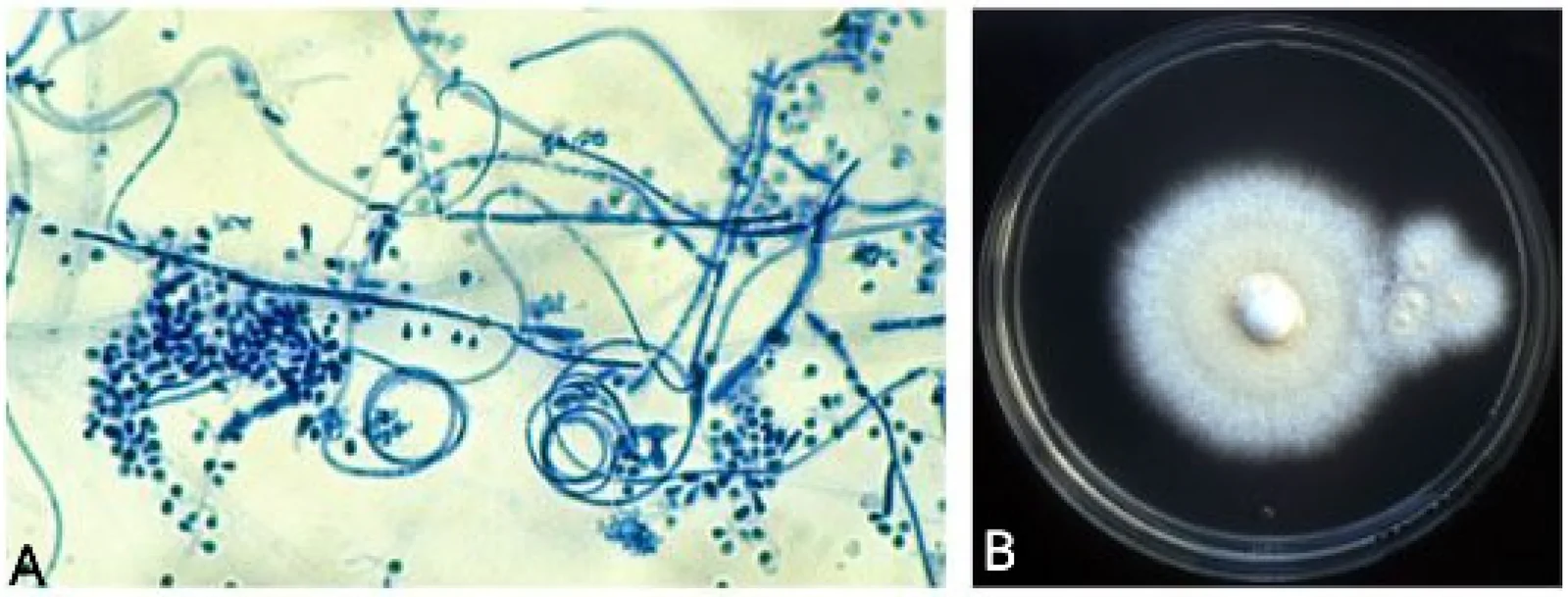
Mycological evaluation of *Trichophyton mentagrophytes* species complex and global epidemiological context. *Trichophyton mentagrophytes* is a cosmopolitan dermatophyte, predominantly zoophilic (commonly associated with rodents, rabbits, and domestic pets, such as ITS genotypes III or VII), though anthropophilic variants (traditionally designated as *T. interdigitale*) also exist. It is currently considered a global public health concern due to the emergence of multidrug-resistant cryptic species (*T. indotineae*) [6,7,8]. The microscopic presentation could equally represent an anthropophilic subtype, such as *Trichophyton interdigitale*. Furthermore, without advanced molecular tools (e.g., ITS sequencing or MALDI-TOF, useful to reach a definitive species-level differentiation), we cannot definitively exclude emerging cryptic species like *T. indotineae*. In our case, microbiological isolation from the periorbital skin and ciliary margin swab was achieved through inoculation on two Sabouraud dextrose agar (SDA) plates (incubated at 37 °C and room temperature) and one dermatophyte test plate (incubated at room temperature) for two weeks. Species identification relied exclusively on these traditional phenotypic criteria. (**A**) Microscopic analysis using lactophenol blue staining revealed numerous unicellular microconidia aggregated in dense, predominantly spherical clusters, along with abundant characteristic spiral hyphae and an absence of macroconidia. (**B**) Macroscopically, fungal cultures yielded white/cream-colored colonies with a powdery surface and raised central tufts, exhibiting a yellow-brown reverse pigmentation.

**Figure 5:**
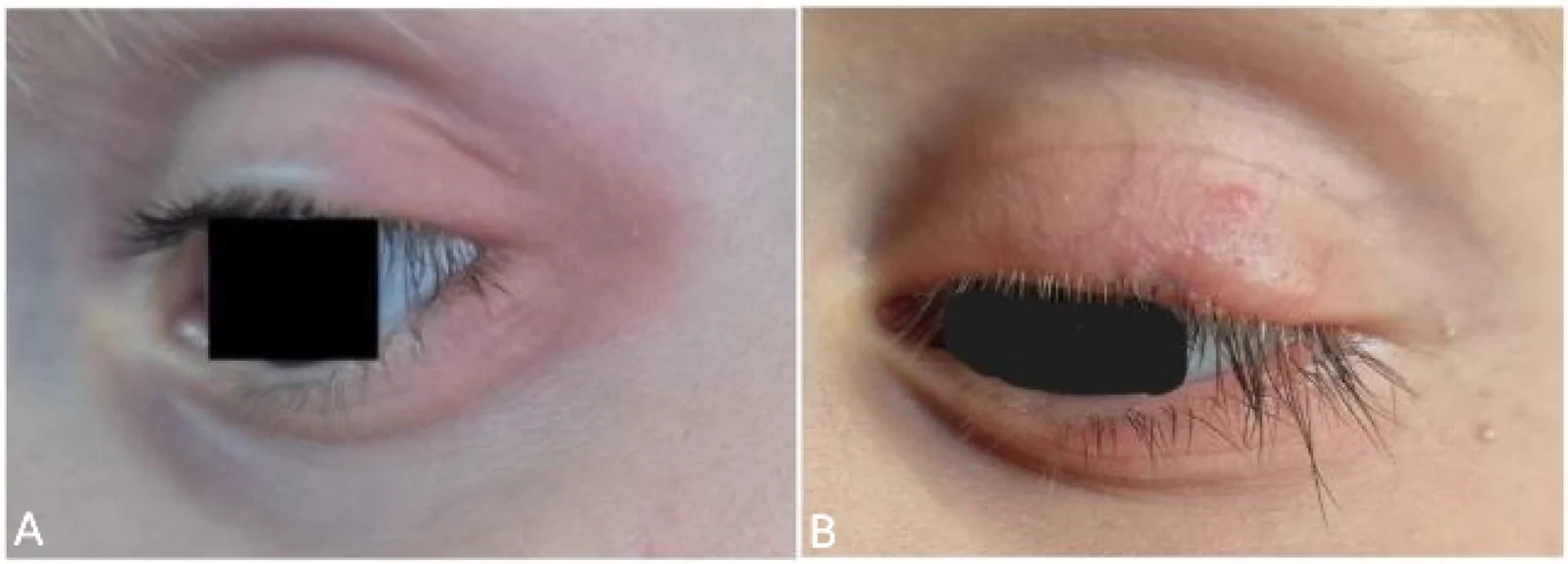
Progressive clinical improvement and near-complete resolution during targeted antifungal therapy. The image illustrates the near-complete clinical resolution of the left periocular lesion during follow-up (**A**) and at the end of the antifungal oral therapy (**B**). The definitive culture result from the initial outpatient swab (subsequently confirmed by the second in-hospital swab) became available on hospital day 7, following an 8-day total incubation period. At this point, clinical improvement was already notable, temporally coinciding with the empiric fluconazole administration. Upon definitive identification of *Trichophyton mentagrophytes* species complex, the broad-spectrum regimen was de-escalated. Therapy was optimized with oral microsize griseofulvin (20 mg/kg/day) and 1% topical terbinafine cream applied twice daily to the periorbital skin for two weeks. Given the close proximity to the eye, explicit instructions were provided to strictly apply the antifungal cream only to the external eyelid skin. Notably, a topical ophthalmic antibiotic ointment was applied to the lid margins prior to the terbinafine cream, acting as a protective barrier to prevent conjunctival toxicity. While emerging terbinafine resistance (e.g., via SQLE gene mutations, mostly associated with *T. indotinae*) within this complex is a growing global concern [9], our decision to avoid terbinafine was primarily driven by pediatric dosing practicalities, regardless of potential susceptibility profiles. In this specific patient, a prudential, extended 12-week course of oral griseofulvin—analogous to *tinea capitis* protocols—was selected due to the deep follicular involvement and the distinct advantage of utilizing a customized galenic liquid formulation, which ensured accurate weight-based dosing and compliance in a 4-year-old child, aiming to support eradication from the hair shafts, minimize the risk of clinical relapse, and allow for the successful, healthy regrowth of the eyelashes. At the 3 months follow-up, sustained clinical resolution was observed. However, a limitation of our management was the lack of post-treatment mycological confirmation via repeat culture or direct examination to definitively prove eradication. Complete mycological clearance requires vigilant follow-up, as these emerging fungal infections are associated with high rates of clinical relapse [2,3,6]. We acknowledge specific limitations in the diagnostic workup of this case. The primary constraint is the absence of advanced molecular biology techniques, such as polymerase chain reaction (PCR) or internal transcribed spacer (ITS) sequencing, which are now considered the gold standard for mycological differentiation. Our identification relied exclusively on traditional phenotypic criteria and macroscopic/microscopic culture evaluation. Consequently, definitive species-level differentiation within the *T. mentagrophytes* complex could not be achieved; this precludes the definitive exclusion of anthropophilic variants (e.g., *T. interdigitale*) or emerging highly virulent cryptic species (e.g., *T. indotineae*). A major diagnostic pitfall in our management was the omission of direct microscopy with KOH. Had this simple point-of-care test been performed initially on skin scrapings or plucked hairs, the 8-day diagnostic delay required for culture incubation could have been significantly shortened. Lastly, the absence of documented direct animal exposure leaves the exact source and transmission route of the infection undetermined. In conclusion, while ‘tinea incognito’ is a well-documented phenomenon, this case provides a stark visual paradigm of how steroid-modified periocular dermatophytosis can clinically mimic a refractory preseptal cellulitis or herpetic infection. The extreme clinical mimicry, which prompted an unnecessary escalation to intravenous broad-spectrum antimicrobials and antivirals, underscores a crucial diagnostic pitfall: the failure to perform early, direct mycological testing. When presumed preseptal infections fail to respond to standard treatments, prompt dermatophyte screening is critical to prevent extensive diagnostic delays and patient morbidity, even in the absence of obvious environmental risk factors.

## Data Availability

The raw data supporting the conclusions of this article will be made available by the authors on request.

## Supplementary Materials

The following supporting information can be downloaded at: https://www.mdpi.com/article/10.3390/diagnostics16203307/s1, Figure S1: Clinical timeline of patient management.

## Abbreviations

The following abbreviations are used in this manuscript:

HSV	Herpes Simplex Virus
