# Supplementary material for: Atypical Periocular Dermatophytosis Caused by Trichophyton mentagrophytes Species Complex Misdiagnosed as Refractory Bacterial and Viral Infection
Source: Diagnostics (Basel). 2026 Oct 12;16(20):3307. doi: 10.3390/diagnostics16203307 (PMC13703535; doi:10.3390/diagnostics16203307)
Supplement: Supplementary file 1 [file diagnostics-16-03307-s001.zip › diagnostics-4581040-supplementary.pdf]

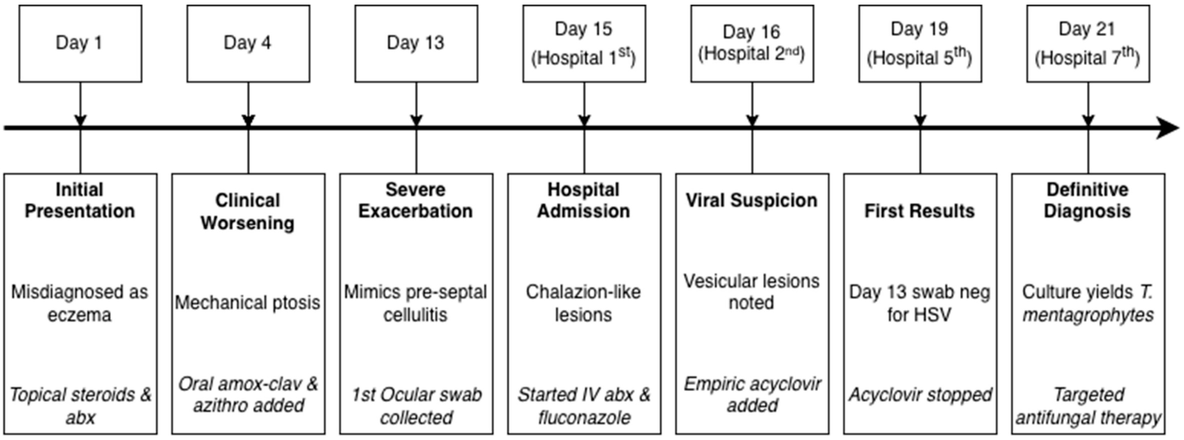


**Figure S1.** Clinical timeline of patient management: The diagram outlines the sequential progression from the initial misdiagnosis and failed empirical regimens to the severe clinical exacerbation. It highlights the critical diagnostic milestones, including the multidisciplinary hospital admission, the viral suspicion, and the definitive mycological identification of *Trichophyton mentagrophytes* species complex that ultimately guided the successful targeted antifungal therapy.
